# 4.1B suppresses cancer cell proliferation by binding to EGFR P13 region of intracellular juxtamembrane segment

**DOI:** 10.1186/s12964-019-0431-6

**Published:** 2019-09-06

**Authors:** Fumin Xue, Chao An, Lixiang Chen, Gang Liu, Feifei Ren, Xinhua Guo, Haibin Sun, Lu Mei, Xiangdong Sun, Jinpeng Li, Youcai Tang, Xiuli An, Pengyuan Zheng

**Affiliations:** 1 Department of Gastroenterology, the Fifth Affiliated Hospital of Zhengzhou University, Zhengzhou, 450052 Henan China; 2 Key Laboratory of H. pylori and Gastrointestinal Microecology of Henan Province, the Fifth Affiliated Hospital of Zhengzhou University, Zhengzhou, 450052 Henan China; 3 Department of Hematology, the First Affiliated Hospital of Zhengzhou University, Zhengzhou, 450052 Henan China; 4 School of Life Sciences, Zhengzhou University, Zhengzhou, 450001 Henan China; 5 Department of Public Health, Zibo Integrate traditional Chinese & Western Medicine Hospital, Zibo, 255000 Shandong China; 6 Red Cell Physiology, New York Blood Center, New York, NY 10065 USA; 7 Department of Pathology, the Fifth Affiliated Hospital of Zhengzhou University, Zhengzhou, 450052 Henan China; 8 Department of Pediatrics, the Fifth Affiliated Hospital of Zhengzhou University, Zhengzhou, 450052 Henan China; 9 Laboratory of Membrane Biology, New York Blood Center, New York, NY 10065 USA

**Keywords:** Gastric cancer, Protein 4.1B, EGFR, Sp1, Tumor suppressor

## Abstract

**Background:**

Gastric cancer (GC) has high incidence and mortality worldwide. However, the underlying mechanisms that regulate gastric carcinogenesis are largely undefined. 4.1B is an adaptor protein found at the interface of membrane and the cytoskeleton. Previous studies demonstrated that 4.1B serves as tumor suppressor.

**Results:**

We showed that 4.1B expression was decreased or lost in most GC patients. The expression pattern of it was tightly correlated with tumor size, TNM stage and overall survival (OS). We further showed that 4.1B inhibited the proliferation of two GC cell lines, MGC-803 and MKN-45, by impeding the EGFR/MAPK/ERK1/2 and PI3K/AKT pathways. A similar phenotype was also observed in immortalized mouse embryonic fibroblasts (MEF) derived from wild type (WT) and 4.1B knock-out (BKO) mice. Additionally, immunofluorescence (IF) staining and Co-IP showed that protein 4.1B bound to EGFR. Furthermore, the FERM domain of 4.1B interacted with EGFR through the initial 13 amino acids (P13) of the intracellular juxtamembrane (JM) segment of EGFR. The binding of 4.1B to EGFR inhibited dimerization and autophosphorylation of EGFR.

**Conclusion:**

Our present work revealed that 4.1B plays important regulatory roles in the proliferation of GC cells by binding to EGFR and inhibiting EGFR function through an EGFR/MAPK/ERK1/2 pathway. Our results provide novel insight into the mechanism of the development and progression of GC.

## Background

Annually, more than 720,000 people die from gastric cancer (GC) worldwide, making it the second leading cause of cancer-related deaths around the world [1]. Half of all GC cases occur in East Asia (mainly in China), of which a total 677,000 cases occur in developing countries [2]. The incidence of GC is 13.9% in total digestive tract and the trend increases with years [3]. Current therapeutic strategies for GC include mucosectomy by endoscopy, gastrectomy, and/or chemotherapy. However, the 5-year overall survival is still only 40% [4]. Therefore, more effective treatment options are urgently needed. Understanding the underlying mechanisms that drive GC pathogenesis will facilitate the development of better therapeutic strategies for GC patients.

Erythrocyte Membrane Protein Band 4.1 Like 3 (EPB41L3 or 4.1B) belongs to the protein 4.1 superfamily, of which there are more than 40 members. This superfamily is classified into five subgroups: Protein 4.1 subfamily, ERM proteins, Talin-related molecules, PTPH (protein tyrosine phosphatases) proteins, and NBL4 (novel band 4.1-like 4) proteins based on protein sequence homology [5]. Protein 4.1B together with 4.1R, 4.1G, and 4.1 N belong to the protein 4.1 subfamily and they are encoded by four paralogous genes [6]. The four members of protein 4.1 subfamily contain four highly conserved functional domains: the membrane-binding FERM domain [7–9], a FERM-adjacent (FA) regulatory domain [10], a spectrin-actin binding domain (SABD) [11, 12], and a C-terminal domain (CTD) unique to the 4.1 proteins [13]. It has been reported that 4.1B is decreased in several cancers, such as renal cell carcinoma [14], ovarian cancer [15], liver cancer [16], and colon cancer [17]. A few studies have addressed the relationship between protein 4.1B and GC [18, 19]; however, details of the functional role of 4.1B in GC development and progression have not been unearthed.

Epidermal growth factor receptor (EGFR) belongs to the Erb receptor tyrosine kinase family, of which there are four members: ErbB1 (EGFR), ErbB2 (c-Neu), ErbB3 (HER3), and ErbB4 (HER4) subtypes). EGFR contains 1186 aa and consists of an extracellular ligand-binding domain, a transmembrane domain, and an intracellular domain. Additionally, the intracellular domain is divided into 3 unique segments, namely the juxtamembrane (JM) segment, tyrosine kinase (TK) domain, and a COOH-terminal region [20–22]. EGFR plays critical roles in regulating metabolism, growth, and differentiation [23]. Numerous studies have documented EGFR overexpression in tumors, such as squamous carcinoma [24] and some glioblastomas [25]. EGFR has potent mitogenic activity that can either stimulate or inhibit growth of a large variety of normal and malignant cells in vitro.

In this article, we report that 4.1B was downregulated in GC specimens and that 4.1B inhibited GC and MEF cell proliferation through the EGFR/MAPK/ERK1/2 and PI3K/AKT pathways. We further demonstrate that 4.1B bound to EGFR through the interaction of the FERM domain of 4.1B with the P13 of EGFR JM segment. Interestingly, this binding blocked EGFR dimerization and autophosphorylation. Our study provides evidence of the molecular mechanism through which 4.1B inhibits cell proliferation.

## Materials and methods

### Mice

Wildtype C57BL/6 mice were purchased from The Jackson Laboratory. The 4.1B knockout mice have been previously reported [26, 27] were kindly provided by Dr. J. Kissil (TheWistar Institute). These mice were housed in the animal facility of New York Blood Center under specific pathogen-free (SPF) conditions accredited by the American Association for Laboratory Animal Care. Four-week-old athymic BALB/c nude mice used for tumorigenicity assays were purchased from the animal center of Beijing, China. They were housed in the animal room of the fifth affiliated hospital of Zhengzhou University under SPF conditions. All animal experiments were approved by the Institutional Animal Care and Use Committee.

### GC cell lines and patient specimens

The GC cell lines MGC-803 and MKN-45 were purchased from Cell Bank of Type Culture Collection of the Chinese Academy of Sciences, Shanghai Institute of Cell Biology. They were cultured in RPMI 1640 medium (HyClone, USA) supplemented with 10% fetal bovine serum (FBS, Invitrogen, USA). All cells were cultured in a humidified incubator at 37 °C and 5% CO_2_.

This study included 102 GC patients who underwent gastrectomy from Jan 2011 to Jan 2013. The normal controls were located at least 5 cm away from the tumor. These GC paraffin-embedded tissue sample collections were approved by the Ethics Committee of the fifth affiliated hospital of Zhengzhou University, Henan, China. The 102 patient specimens included 59 males and 43 females. The patient age range was from 25 to 76 years old. Histological grades were classified according to the WHO classification as highly (grade I), moderately (grade II), and poorly differentiated (grade III). The TNM stage was performed according to the AJCC8 classification.

### Immortalized MEF cells preparation and culture

Isolation of primary mouse embryo fibroblasts (MEF) from day 13.5 embryos (E13.5) of 4.1B^+/+^ and 4.1B^−/−^ C57BL/6 mice was performed as previously described [28]. Briefly, the head and internal organs were removed. The remaining embryonic tissues were minced using a pair of scissors and immersed in 0.25% trypsin overnight at 4 °C. After 24 h, MEFs were collected after centrifugation at 1500 rpm and maintained in Dulbecco Modified Eagle Medium (DMEM) containing 10% fetal bovine serum (FBS, GIBCO, USA) and 100 μg/ml penicillin/streptomycin (LEAGENE, China). After two passages, the MEFs were immortalized by retroviral transduction of the SV40 large T antigen. MEFs were cultured in DMEM containing 10% FBS and incubated at 37 °C in a humidified environment with 5% CO^2^.

### Cloning of 4.1B cDNA from MEF cells

Total RNA was isolated from wild type and 4.1B knock-out MEF cells by the RNeasy mini kit (Qiagen, Germany). 1 μg of RNA was reverse-transcribed into cDNA using random primers and M-MuLV reverse transcriptase (New England Biolabs, USA). Reverse transcription was performed according to the manufacture instructions (New England Biolabs, USA). Primers used to amplify 4.1B transcripts were: 4.1B ATG-1 FOR: 5′-ATGACGACCGAATCAGGATCAGACTCAG-3′; 4.1B RE: 5′-TCAATCCTCTCCGTCCTCTGGTGTGATT-3′. PCR was performed in a 50 μl reaction mixture containing 2 × Hotstar Taq Plus Master Mix DNA Polymerase (Qiagen, Germany), 10 μM primer each, 200 ng of template cDNA, and double distilled H_2_O. Cycling conditions were 30 s at 94 °C for denaturation, 30 s at 55 °C,4 min at 68 °C for extension, and a final extension for 5 min at 72 °C. Cycle numbers were 45 for ATG-1 4.1B.

### Plasmid constructions

For mammalian cell expression, ATG-1-4.1B was cloned into pEGFP-C3 vector using restriction enzymes XhoI and BamHI upstream and downstream, the primers used were:4.1B forward 5′-AAACTCGAGATGACGACCGAATCAGGATCAGACTCAG-3′; 4.1B reverse 5′-AAAGGATCCCGTCAATCCTCTCCGTCCTCTGGTGTGATT-3′. The 4.1B 130-kDa protein was cloned into pET-31b (+) with NsiI and XhoI upstream and downstream, respectively. The various domains of 4.1B were cloned into pGEX4T-2 or pET28c vector. The EGFR intracellular fragments EGFR1, EGFR2 and the EGFR1 without 13 amino acid fragment (EGFR1△aa13) were cloned into pGEX4T-2 vector; using restriction enzymes SmaI and XhoI upstream and downstream.

The primers used as follow: the first fragment of intracellular EGFR (EGFR1) forward 5′ ATCCCGGGCGAAGACGTCACATTGTTCGA 3′; the first fragment of intracellular EGFR (EGFR1) deletion the initial 13 amino acid (EGFR1△aa13) forward 5′-ATCCCGGGCTGCTTCAAGAGAGAGAGCTC-3′; the first fragment of EGFR (EGFR1) reverse 5′-ATCTCGAGCGTCAAACAAGGTAGCGCTGTGGGTC-3′; the second fragment of intracellular EGFR (EGFR2) forward 5′-ATCCCGGATCCAGGGGGATGAAAGAATG-3′; the second fragment of intracellular EGFR (EGFR2) reverse 5′-CGCTCGAGCGTCATGCTCCAATAAACTCACT-3′. Synthesizing of polypeptide of EGFR P13 amino acid and mutants of P13 amino acid, the EGFR polypeptide used were: EGFR P13-RRRHIVRKRTLRR, The mutants of EGFR P13 (RRR-*AAA*): *AAA*HIVRKRTLRR; EGFR P13 (RKR-*AAA*): RRRHIV*AAA*TLRR; EGFR P13 (RR-*AA*): RRRHIVRKRTL*AA*.

### Immunofluorescence

For confocal immunofluorescence microscopy assay, cells were grown on Lab-Tek™ Chambered Coverglass (Thermo Fisher Scientific, USA) pre-coated with 10 μg/ml fibronectin (Gibco, USA). Cells were fixed with 1% paraformaldehyde (LEAGENE, China) for 15 min and then permeabilized with 0.1% Triton X-100 (Sigma-Aldrich, USA) in 0.25% paraformaldehyde-PBS for 15 min at room temperature, followed by blocking in 10% horse serum (Gibco, USA), 0.1% Triton X-100 in PBS for 30 min to minimize nonspecific antibody binding. Cells were then incubated with primary antibodies at 4 °C overnight and then washed with PBS 3 times. This was followed by incubation with the appropriate secondary antibody at room temperature for 30 min. The secondary antibodies were donkey anti-rabbit (Thermo Fisher Scientific, USA) and donkey anti-mouse IgG (Thermo Fisher Scientific, USA) labeled with Alexa Fluor 488 or Alexa Fluor 594. To-pro-3 (Invitrogen, USA) was used to stain the nucleus. Images were collected on a Zeiss LSM510 META confocal microscope (Germany) using × 63 oil-immersion objective.

### Cell proliferation assay

5 × 10^5^ cells were seeded in the cell culture dish and counted on day 3, day 5, and day 7. The untreated MGC-803 and MKN-45 cells were cultured in RPMI-1640 with 10% FBS. The transfected MGC-803 and MKN-45 cells underwent selection with G418 (Gibco, USA) and puromycin (Millipore, USA). The experiment was independently repeated three times.

### Cell transfection

MGC-803 and MKN-45 cells were seeded in six-well plates with RPMI-1640 with 10% FBS. When cells reached 80–85% confluence, MGC-803 cells were transfected with 3 μg/ml pEGFP-4.1B or pEGFP-C3 plasmid using lipofectamine 2000 (Invitrogen, USA) following the manufacturer’s instructions. Stably transfected cells were obtained by selection with 400 μg/ml G418 for 24 h. To knockout 4.1B, Plasmid 4.1B double nickase plasmid (sc-406,853-NIC) and control nickase plasmid (sc-437,281) were used to transfect MKN45 cells. Stably transfected cells were obtained after selection with 8 μg/ml puromycin for 36 h.

MKN-45 4.1B−/− cells and 4.1B KO MEF cells were transfected with small interfering RNAs (siRNAs) (100 nM) against Sp1 which were purchased from GenePharma (China, shanghai). All siRNAs were transfected using lipofectamine 2000 following the manufacturer’s instructions. The knockdown efficiencies were detected by Real-time PCR and western blotting after 48 h. The sequences of siRNAs were as follows: human and mouse negative control FAM 5′ – UUCUCCGAACGUGUCACGUTT – 3′; human Sp1 5′ – CCAUUAACCUCAGUGCAUUTT – 3′; mouse Sp1 5′ –GCGGCAAAGUAUAUGGCAATT– 3′.

### Western blot analysis

Cells were lysed with RIPA buffer (150 mM NaCl, 25 mM Tris-HCl pH 7.4, 0.1% SDS, 1% Triton X-100, 1% deoxycholate, 2 mM EDTA, Millipore, USA) supplemented with protease inhibitor (Sigma-Aldrich, USA) and phosphatase inhibitor (Roche, Basel, Switzerland) for 30 min on ice. The supernatant was collected by centrifugation with 14,000 rpm and the protein concentration was measured with BCA kit (Thermo Fisher Scientific, USA). Protein samples (50 μg) were separated by 10% SDS-PAGE gels and transferred to nitrocellulose membranes. The membranes were incubated with primary antibodies overnight at 4 °C. Membranes were then washed with TBS-T and then incubated with secondary antibodies for 1 h. Signals were detected with ECL kit (Thermo Fisher Scientific, USA) by Chemi Doc™ XRS+ with Image Lab™ Software (Bio-Rad, USA).

The primary antibodies included 4.1B(HP) which was characterized and used in our previously published studies [29], p-EGFR (Cell Signaling Technology, USA, #3777), EGFR (Cell Signaling Technology, USA, #2232), p-ERK1/2 (Abcam, UK, ab50011), ERK1/2 (Abcam, UK, ab17942), p-AKT (Cell Signaling Technology, USA, #4060), AKT (Cell Signaling Technology, USA, #4691), p-JNK (Abcam, UK, ab76572), JNK (Abcam, UK, ab208035), p-p38 (Abcam, UK, ab178867), p-38 (Abcam, UK, ab27980), Sp1 (Santa Cruz Biotechnology, CA, sc-17,824), β-actin (Santa Cruz Biotechnology, CA, sc-47,778).

### Immunohistochemistry

The paraffin-embedded patient specimens were sectioned at 3 μm thickness and mounted on glass slides. After deparaffinization and dehydration with xylene and an alcohol gradient, the slides were washed with PBS. Antigen retrieval was carried out by heating slides for 20 min in citrate buffer (LEAGENE, China). Endogenous peroxidase activity was blocked with 3% hydrogen peroxide and 10% goat serum (Beijing Zhongshanjinqiao Biotechnology Co. LTD, China) for 30 min at room temperature. The slides were incubated with an anti-4.1B primary antibody at 4 °C overnight. Biotinylated goat anti-rabbit IgG antibody was then added to the slides for 30 min at 37 °C after washing with PBS. Diaminobenzidine (Beijing Zhongshanjinqiao Biotechnology Co. LTD, China) was used to stain the slides for about 1 to 5 min. Slides were then counterstained with hematoxylin (LEAGENE, China) and dehydrated in xylene and mounted with permount.

The result was assessed by an addition of the percentage of positive tumor cells and the cytoplasmic staining intensity. The percentage of positive tumor cells was scored as 0 (< 5%), 1 (5–25%), 2 (25–50%), 3 (50–75%), or 4 (> 75%). The staining intensity was scored as 0 (negative), 1 (weak), 2 (moderate), or 3 (strong). A sample was defined as negative if the final score was 0–3 and as positive if the final score was 4–7. *P* values were calculated using the chi-squared test.

### Tumorigenicity analysis

Five-week-old BALB/C nude mice were injected with 3 × 10^6^ of each transfected cell type suspended in 200 μl serum-free RPMI-1640. The cells were inoculated into the subaxillary region for the tumorigenic assay. Tumors were palpable after 1 week and monitored every 3 days. The tumor size was measured with a Vernier caliper and tumor volume was calculated as follows: volume = 1/2 × (long axis) × (short axis)^2^. Experimental mice were euthanized after 2 weeks. The resected tumors were fixed with 10% formalin, embedded in paraffin, and sectioned (3 μm). Sections were stained with hematoxylin and eosin (H&E) (LEAGENE, China) and Ki-67 (LEAGENE, China) for light microscopy examination.

### RNA isolation, reverse transcription, and PCR analysis

Total RNA was isolated from wild type and 4.1B knock-out MEF cells, MGC-803 pEGFP-C3 control and pEGFP-4.1B overexpression cells by total RNeasy mini kit (Qiagen, Germany). 1 μg of total RNA from each sample was reverse-transcribed into cDNA following the manufacturer’s protocol (TOYOBO, Japan). The primers used for PCR were as follows: human 4.1B forward, 5’- CTAGCAGTAAACTCTCTCGGTCT − 3′ and reverse 5′- TGGAGCGTTTCTCTACATCACA − 3′; human EGFR forward, 5′- TTGCCGCAAAGTGTGTAACG − 3′ and reverse 5′- GTCACCCCTAAATGCCACCG − 3′; human SP1 forward, 5′-AAGTAATCCCACAGTTCCAGACC − 3′ and reverse 5′- GTTGGTTTGCACCTGGTATGATC-3′; human GAPDH forward, 5′- AGAAGGCTGGGGCTCATTTG − 3′ and reverse 5′- AGGGGCCATCCACAGTCTTC − 3′; mouse 4.1B forward, 5’- AAGAGCCACAGAGGAATGACG − 3′ and reverse 5′- TCCTTGGCATGGTGTAAGTCC − 3′; mouse EGFR forward, 5′- AATGTTCCCATCGCTGTCGT − 3′ and reverse 5′- GGCAGACCAGACAGTCACTC − 3′; mouse SP1 forward, 5′- TACCACCCTAACACCCATTGC − 3′ and reverse 5′- TCCCTGAAGTACCCAATGCAC-3′; mouse β- actin forward, 5′- GCTTCTTTGCAGCTCCTTCGT − 3′ and reverse 5′- CCAGCGCAGCGATATCG -3′. PCR was performed following the manufacturer’s instructions (TOYOBO, Japan). The reaction mixture included 25 μl SYBR Green Real-time PCR Master Mix, 0.4 μM primer each, 5 μl of template cDNA, and double distilled H2O and total volume is 50 μl. And cycling conditions were 60 s at 95 °C for denaturation, 15 s at 60 °C or annealing, 45 s at 72 °C for extension. Cycle numbers were 40. The results were collected with AriaMx Real-time PCR system (Agilent Technologies, USA).

### Co-immunoprecipitation

MEFs were lysed with ice-cold lysis buffer (50 mM HEPES, pH 8.3, 420 mM KCl, 0.1% NP-40, 1 mM EDTA) supplemented with a proteinase inhibitor cocktail (Roche, Basel, Switzerland) for 30 min on ice. Supernatants were collected after centrifugation at 15,000 g at 4 °C for 10 min and protein concentration was determined by the Bradford method using BSA as a standard (Bio-Rad, USA). Protein extracts (500 μg per sample) were incubated with either 5 μg anti-4.1B-HP or anti-EGFR antibody or pre-immune IgG in 500 μl of Co-IP buffer (Active motif, CA) at 4 °C overnight with rotation. The immunoprecipitates were isolated using Protein-G beads (Millipore, USA) and separated by 10% SDS-PAGE and then transferred to a nitrocellulose membrane. The membrane was probed with antibodies against EGFR or 4.1B-HP.

### Pull-down assay

GST-tagged recombinant different fragment cytoplasmic domain of EGFR and various functional domains of 4.1B were coupled to glutathione-Sepharose-4B beads at room temperature for 30 min. Immobilized streptavidin beads were mixed with biotinylated 13 EGFR peptide prior to incubation with GST-tagged 4.1B domains. Beads were pelleted and washed. His-tagged 4.1B with different GST-tagged cytoplasmic domain of EGFR and GST-tagged 4.1B domains with biotinylated 13 EGFR peptide were added to the coupled beads in a final volume of 100 μl. The final concentration of the coupled protein was 2 μM. The mixture was incubated for 1 h at room temperature, pelleted, washed and eluted with 10% SDS. The pellet was analyzed by SDS-PAGE. The binding of His-tagged 4.1B domains with EGFR intracellular fragments was detected by western blot using anti-His antibody. The binding of GST-tagged 4.1B domains with 13 EGFR peptide was detected by western blot using anti-GST antibody. GST was used as negative control in all experiments.

### Statistics

All statistical analyses were performed by using SPSS 20.0 software. Difference between 2 groups was determined by Student t test orχ^2^ test. DFS and OS were analyzed by Kaplan-Meier analysis with the log-rank test applied for comparison. And the survival data were examined by univariate and multivariate COX proportional hazards model. Variables with a value of *p* < 0.05 in univariate were checked in multivariate analysis by COX regression analyses. And two-sided *p* < 0.05 was considered statistical significance.

## Results

### Expression of 4.1B in GC patients and GC cell lines

**Fig. 1:**
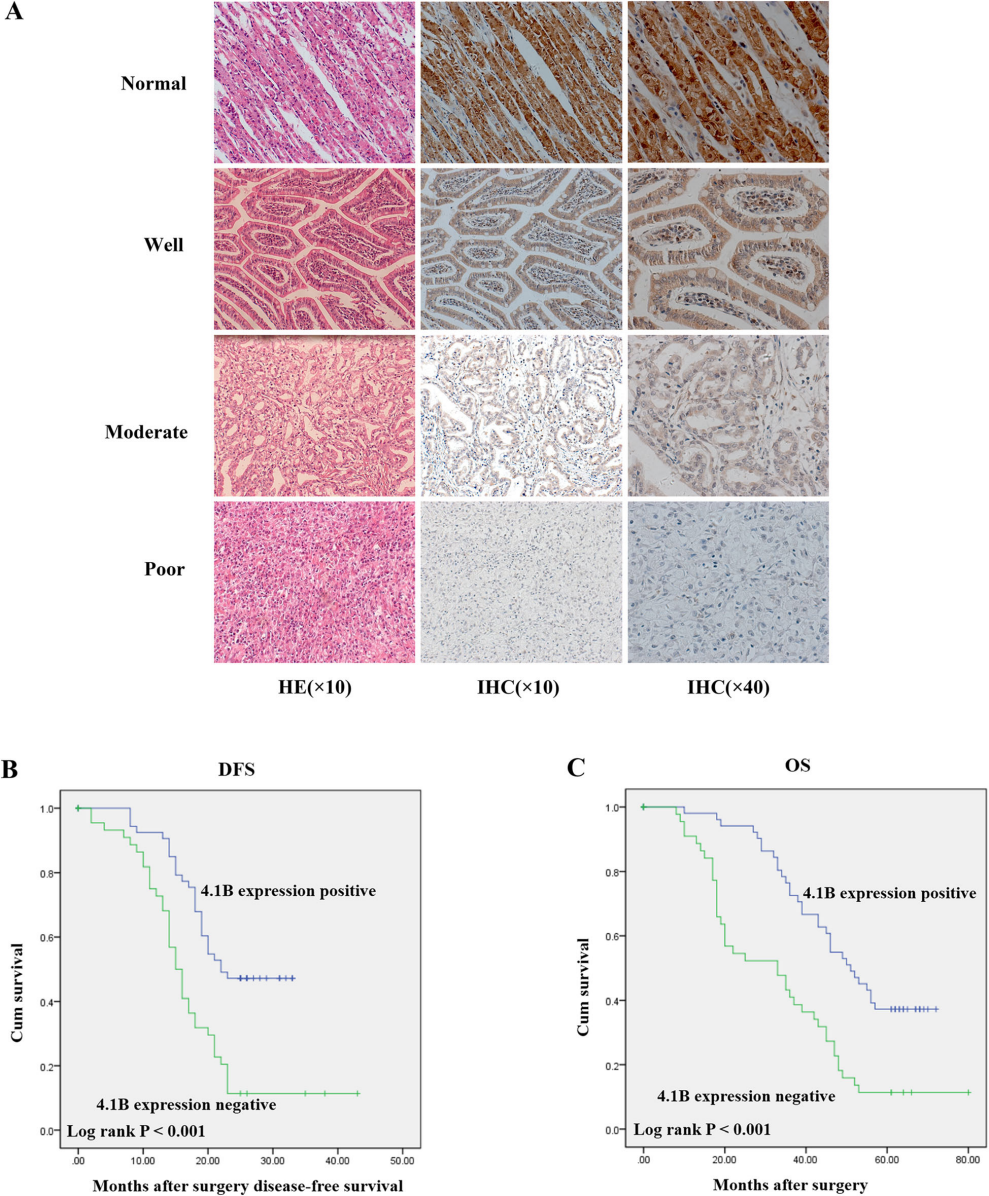
4.1B expression levels in GC primary tumor and the correlation with prognosis. **a** Representative images of immunohistochemical staining for protein 4.1B expression. Normal gastric tissues (adjacent noncancerous gastric tissues of GC specimens (> 5 cm)) and GC specimens (well, moderate and poor) were stained for 4.1B by an anti-4.1B HP antibody. The brown color represents positive staining for 4.1B expression. Cells nuclei were counterstained with hematoxylin (blue). The purple color staining is HE staining. Original magnification: × 10 and × 40. **b** Disease-free survival was analyzed by Kaplan-Meier analysis in GC patients with 4.1B protein positive (score 4–7) and negative (score 0–3) specimens. **c** Overall survival was analyzed by Kaplan-Meier analysis in GC patients with 4.1B protein positive (score 4–7) and negative (score 0–3) specimens

To explore the relationship between 4.1B and GC, we analyzed 4.1B expression in 102 gastric adenocarcinoma patient samples by immunochemistry. The results revealed that 4.1B was significantly different between tumor tissues and their matched adjacent non-tumor tissues (Fig. 1a; Table 1; *χ*^2^ = 32.23; *p* < 0.001). There was strong expression of 4.1B in the cell membrane and cytoplasm of the majority of adjacent non-tumor tissues (91.17%) (Fig. 1a). In contrast, tumor tissues showed little to no expression of 4.1B (Fig. 1a). There was a negative correlation between 4.1B expression level with tumor size, pathologic differentiation, lymph node metastasis and TNM stage (Table 2). There was no significant association between 4.1B expression and patients age, gender, *Helicobacter pylori* infection, tumor location, WHO classification, vascular invasion, depth of invasion and neural invasion (Table 2).

**Table 1:** The difference of 4.1B expression in GC and adjacent normal tissue specimens

Variables		gastric adenocarcinoma		Total	χ^2^	*p* value
		negative	positive			
Adjacent normal tissue	negative	8	1	9		
	positive	38	55	93	32.230	< 0.001
Total		46	56	102

**Table 2:** Correlation of 4.1B expression with clinical parameters in GC patients

Variables	No.(*n* = 102)	4.1Bexpression		χ^2^	*p* value
		Negative(46)	Positive(56)		
Age (years)					
<65	49	21	28	0.191	0.662
≥ 65	53	25	28		
Gender					
Male	59	27	32	0.025	0.847
Female	43	19	24		
Helicobacter pylori infection					
Positive	56	24	32	0.252	0.616
Negative	46	22	24		
Tumor size					
<5 cm	56	19	37	6.257	0.012*
≥ 5 cm	46	27	19		
Tumor location					
Cardia	26	6	20	6.956	0.073
body	26	13	13		
Antrum	41	22	19		
Whole	9	5	4		
WHO classification					
Tubular	32	10	22	5.281	0.152
Signet-ring cell	29	14	15		
Mucinous	37	21	16		
Others	4	1	3		
Pathologic differentiation					
G1	14	4	10	8.025	0.018*
G2	35	11	24		
G3	53	31	22		
Vascular invasion					
Negative	42	15	27	2.539	0.111
Positive	60	31	29		
Neural invasion					
Negative	43	17	26	0.929	0.335
Positive	59	29	30		
Depth of invasion					
T1 + T2	26	8	18	2.894	0.089
T3 + T4	76	38	38		
Lymph metastasis					
N0	26	7	19	4.656	0.031*
N1 + N2 + N3	76	39	37		
TNM stage					
I	19	3	16	8.589	0.014*
II	26	12	14		
III	57	31	26		

* *p* < 0.05

To further explore the correlation of 4.1B and prognosis, we analyzed the median survival about the disease-free survival (DFS) and overall survival (OS) by Kaplan-Meier analyses and log-rank test. Seven specimens were excluded because of accidental death or lost to follow-up. The DFS results showed the 5-year median survival of 4.1B positive patients was 22 months, while the 4.1B negative specimens was 15 months. The 5-year median survival about OS is 51 months in 4.1B positive patients, while the 4.1B negative specimens was 33 months. This result showed that 4.1B positive GC patients had better survival time than negative ones (Fig. 1b and c; *p* < 0.001).

To further evaluate whether 4.1B is an independent factor for GC patient prognosis, we performed the univariate and multivariate COX regression analyses for DFS and OS. The results showed the 4.1B expression, tumor size, lymph metastasis (N0 vs. N1 + N2 + N3) and TNM analyses (I + II vs. III) were the prognostic factor. Further analysis in the multivariate COX regression analyses, 4.1B expression, tumor size and TNM stage were the independent factor for DFS (HR:1.773, CI:1.036–3.035, *P* = 0.037; HR:1.694, CI:1.023–2.803, *P* = 0.040 and HR:1.923, CI:1.161–3.184, *P* = 0.011) and OS (HR:1.691, CI:1.013–2.823, *P* = 0.045; HR:2.025, CI:1.242–3.302, *P* = 0.005 and HR:2.197, CI:1.365–3.534, *P* = 0.001) (Table 3).

**Table 3:** Univariate and multivariate COX regression analyses 4.1B for DFS or OS in GC patients

Variables	DFS			OS		
	HR	95% CI	p value	HR	95% CI	*p* value
Univariate analysis						
4.1B expression (Positive vs. Negative)	2.654	1.626–4.332	< 0.001*	2.547	1.584–4.095	< 0.001*
Age (< 65 years vs. ≥ 65 years)	1.092	0.676–1.766	0.719	0.879	0.551–1.400	0.586
Gender (male vs. female)	0.910	0.558–1.482	0.704	0.953	0.593–1.530	0.841
HP infection (Positive vs. Negative)	1.131	0.699–1.832	0.616	1.161	0.726–1.857	0.533
Tumor size (<5 cm vs. ≥ 5 cm)	1.993	1.230–3.230	0.005*	2.260	1.403–3.640	0.001*
Tumor location (Cardia+body vs. Antrum +Whole)	1.413	0.872–2.289	0.160	1.374	0.861–2.194	0.183
WHO classification (Tubular +Signet-ring cell vs. Mucinous+ Others)	1.247	0.770–2.022	0.369	1.128	0.705–1.805	0.616
Pathologic differentiation (G1 + G2 vs. G3)	1.229	0.867–1.742	0.247	1.066	0.771–1.473	0.699
Vascular invasion (Positive vs. Negative)	1.229	0.752–2.010	0.411	1.142	0.709–1.840	0.584
Neural invasion (Positive vs. Negative)	1.213	0.748–1.968	0.433	1.288	0.804–2.064	0.293
Depth of invasion (T1 + T2 vs. T3 + T4)	1.429	0.814–2.508	0.214	0.938	0.563–1.563	0.807
Lymph metastasis (N0 vs. N1 + N2 + N3)	2.266	1.211–4.238	0.010*	1.980	1.117–3.512	0.019*
TNM stage (I + II vs. III)	2.317	1.428–3.760	0.001*	2.578	1.657–4.011	< 0.001*
Multivariate analysis						
4.1B expression (Positive vs. Negative)	1.773	1.036–3.035	0.037*	1.691	1.013–2.823	0.045*
Tumor size (<5 cm vs. ≥ 5 cm)	1.694	1.023–2.803	0.040*	2.025	1.242–3.302	0.005*
Lymph metastasis (N0 vs. N1 + N2 + N3)	1.724	0.903–3.289	0.099	1.458	0.807–2.632	0.211
TNM stage (I + II vs. III)	1.923	1.161–3.184	0.011*	2.197	1.365–3.534	0.001*

* *p* < 0.05

**Fig. 2:**
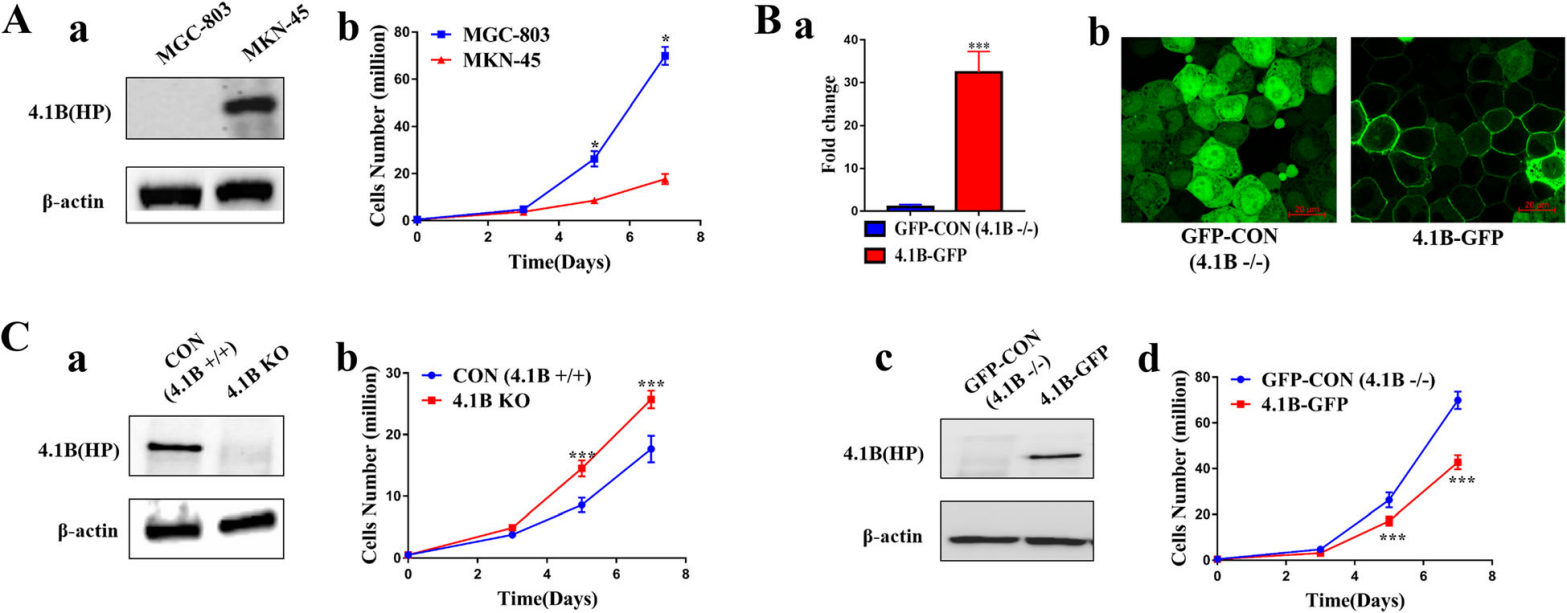
Effects of 4.1B on the proliferation of transfected GC cell lines in vitro. **a** 4.1B expression in MGC-803 and MKN-45 GC cell lines (a) and their proliferation ability (b). **b** mRNA level and localization of 4.1B was examined by Real-time PCR (a) and confocal microscopy (b) in 4.1B-overexpressing MGC-803. Scale bars, 20 μm. 4.1B expression in 4.1B-overexpressing MGC-803 cell lines (c) and their proliferation ability change (d). **c** 4.1B expression in 4.1B KO MKN-45 GC cell lines (a) and their proliferation ability change (b). * *p* < 0.05, ** *p* < 0.01, *** *p* < 0.001

We also investigated 4.1B protein expression in two GC cell lines with starkly different rates of proliferation (Fig. 2A). There was a clear detection of a prominent protein band at 130 kDa in the MKN45 cell line. However, no such band was detected in the MGC-803 cell line (Fig. 2A). The mRNA level of 4.1B was also consistent with the protein result by real-time PCR (data not shown). These results suggest that 4.1B was inversely associated with the proliferation of GC cell lines.

### 4.1B suppresses GC cell proliferation in vitro

To further explore the effect of 4.1B in GC cell lines, we transiently transfected MGC-803 cells with pEGFP-4.1B to exogenously increase 4.1B expression. Conversely, 4.1B double nickase plasmid was transfected transiently to silence the expression of endogenous 4.1B. Real-time PCR and western blot analysis confirmed successful overexpression at the mRNA and protein level and knockout at protein level (Fig. 2B-a and b; C-a).

Figure 2B-d showed that 4.1B overexpression suppressed proliferation of MGC803 in comparison to pEGFP-C3 control at different time points (*p* < 0.001). Conversely, proliferation was promoted in 4.1B-silenced MKN45 cells (Fig. 2C-b; *p* < 0.001).

Collectively, these results demonstrated that 4.1B can suppress GC cell proliferation. These results suggest that 4.1B can act as a tumor suppressor gene in GC cells.

### 4.1B suppresses tumor growth in vivo

**Fig. 3:**
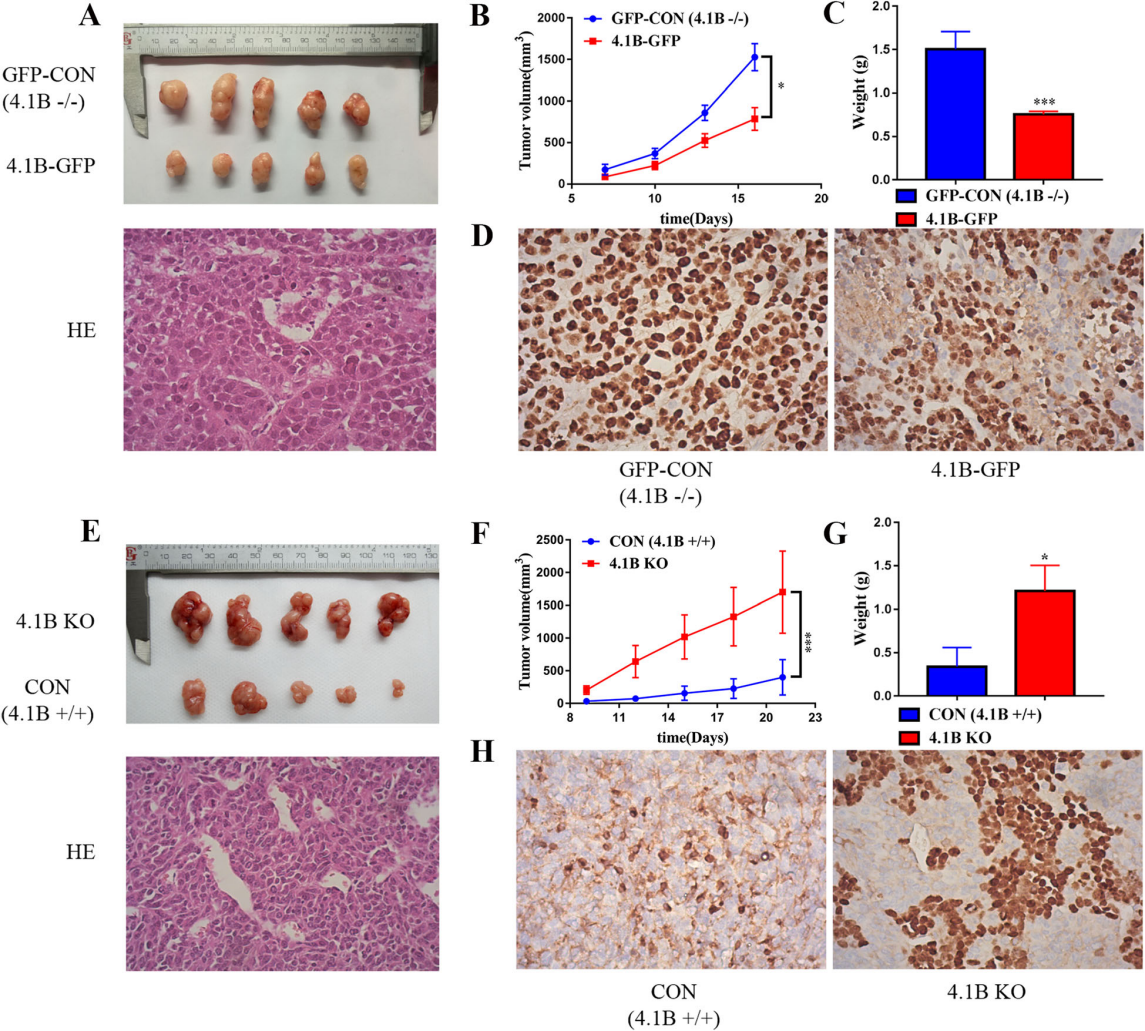
Anti-tumor effects of 4.1B were evaluated in the nude mice. **a- d** Effects of 4.1B overexpression on tumor growth in the subcutaneous xenograft model. **e**- **h** Effects of 4.1B knock out on tumor growth in the subcutaneous xenograft model. **a**, **e** Images of stripped tumors from mice (*n* = 5 mice for each group). **b**, **f** Tumor growth curves showed that 4.1B overexpression reduced the growth of xenografts in nude mice, and 4.1B knock out promoted the growth of xenografts in nude mice. The values at each time point are shown as mean tumor size (mm3) ± SEM (standard error of the mean). **c**, **g** The weight of xenograft tumors at the time of sacrifice. Values are shown as mean tumor weight (**g**) ± SEM. **d**, **h** Ki-67 expression in xenograft tumors. * *p* < 0.05, ** *p* < 0.01, *** *p* < 0.001

To further examine the role of 4.1B in tumor progression in vivo, we injected the four cell lines (MGC-803 pEGFP-4.1B (4.1B +/+), pEGFP-C3 control (4.1B −/−) and MKN-45 4.1B KO (4.1B −/−), NIC control (4.1B +/+)) subcutaneously into the right subaxillary region of nude mice. Tumors were visible after 1 week, and the size of tumors was measured every other day by a Vernier caliper. All the mice were sacrificed when the maximum diameter reached 20 mm. Results showed tumorigenic ability was weakened in 4.1B-overexpressing MGC-803 cells. Tumor volume was smaller than control MGC-803 GFP-only cells (4.1B −/−) (Fig. 3a and b; *p* < 0.001). Subcutaneous injection of MKN-45 cells with 4.1B gene depletion resulted in larger tumors than the NIC control (4.1B +/+) (Fig. 3e and f; *p* < 0.001). Tumor weight was consistent with tumor volume. Tumors from 4.1B-deficient cells were heavier than tumors from 4.1B-sufficient cells (Fig. 3c and g; *p* < 0.001and *p* < 0.05). The mitotic ability, as shown by Ki-67 staining, was suppressed in 4.1B-sufficient cells (Fig. 3d, h).

### 4.1B suppresses GC cell proliferation by affecting the EGFR/MAPK/ERK1/2 and PI3K/AKT pathways

Previous studies have focused on the relationship between 4.1B and cell differentiation [17], adhesion [30], motility [31], and apoptosis [32]. There were also some studies on 4.1B and cell proliferation, but these studies were limited to a characterization of cell cycle proteins [33]. We wanted to further explore the mechanisms by which 4.1B affects cell proliferation.

**Fig. 4:**
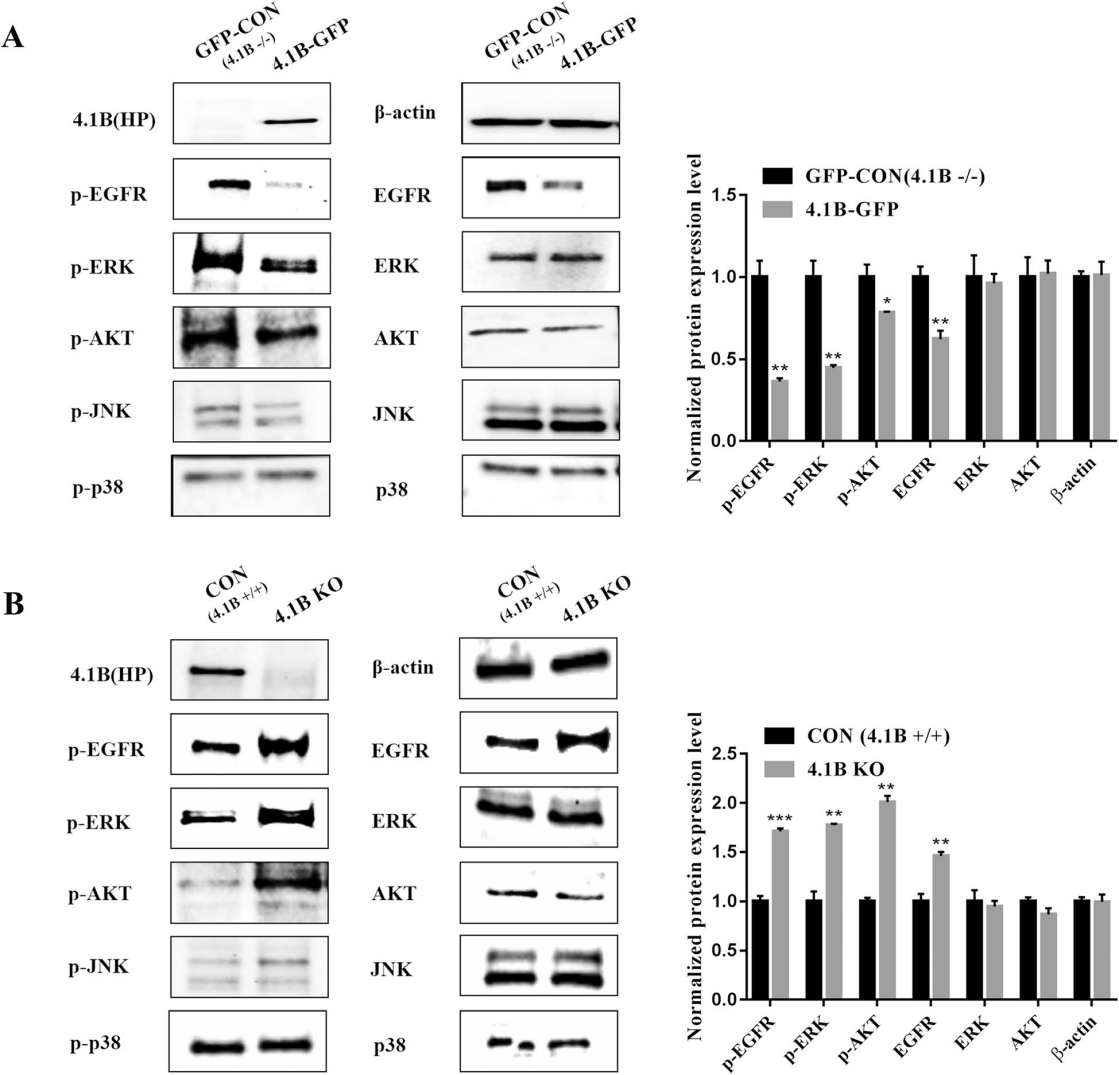
Effects of 4.1B on signaling transduction pathway in GC cell lines (MGC-803 and MKN-45). **a** Phosphorylation of indicated molecules in GFP or GFP-4.1B -overexpressing MGC-803 cell lines. β-actin served as the loading control. **b** Phosphorylation of indicated molecules in MKN-45 or 4.1B KO MKN-45 cells. β-actin served as the loading control. * *p* < 0.05, ** *p* < 0.01, *** *p* < 0.001

Cell proliferation is commonly regulated by the EGFR/MAPK and PI3K/AKT pathways. Therefore, we examined changes in these two key signaling pathways by western blot. Figure 4 showed that 4.1B deficiency resulted in markedly increased levels of ERK1/2 and AKT phosphorylation. Total ERK1/2 and AKT protein levels remained unchanged. 4.1B did not affect p38 and JNK phosphorylation or their corresponding total protein levels. Interestingly, we observed that 4.1B loss resulted in an increase in the protein level of EGFR (Fig. 4).

### Deficiency of 4.1B leads to hyperproliferation of MEF cells due to increased EGFR expression

**Fig. 5:**
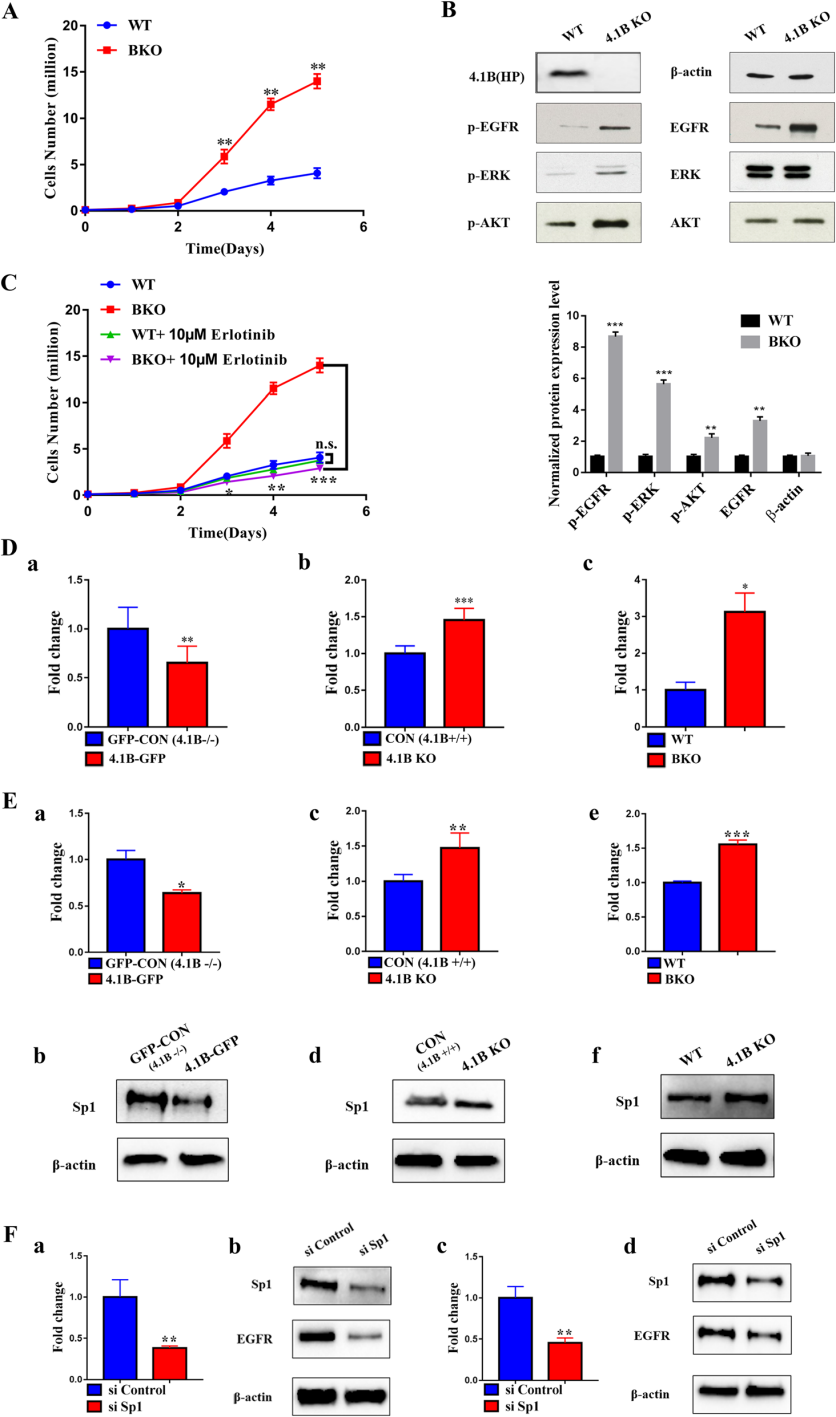
Hyperproliferation and enhanced EGFR-mediated signal transduction of immortalized 4.1B KO MEF cells. **a** Proliferation of immortalized WT MEF and 4.1B KO MEF. **b** Increased phosphorylation of EGFR, ERK and AKT in 4.1B KO MEF cells. β-actin served as the loading control. **c** Effects of erlotinib on cell proliferation. **d** Expression of EGFR mRNA levels in various cell types as indicated: GFP or GFP-4.1B -overexpressing MGC-803 cell lines (a); MKN-45 or 4.1B KO MKN-45 cells (b); immortalized WT MEF and 4.1B KO MEF (c). **e** Decreased expression of Sp1 mRNA and protein levels in 4.1B overexpressing MGC-803 cells (a; b) and increased expression of Sp1 mRNA and protein levels in 4.1B-deficient MKN-45 cells (c; d) and 4.1B KO-MEF cells (e; f). **f** Decreased expression of EGFR in Sp1 knockdown cells (4.1B-deficient MKN-45 cells mRNA level (a) and protein level (b)); (4.1B KO-MEF cells mRNA level (c) and protein level (d)). * *p* < 0.05, ** *p* < 0.01, *** *p* < 0.001

We then examined the proliferation of immortalized MEFs derived from wild type and 4.1B knockout mice. Figure 5A shows increased proliferation of 4.1B knock-out MEF cells. The increased proliferations were accompanied by the increased phosphorylation of ERK1/2, AKT and EGFR (Fig. 5B). Interestingly, while there were no changes in protein levels of ERK1/2 and AKT, total EGFR protein level was also upregulated in immortalized 4.1B KO MEFs (Fig. 5B). These findings suggest that 4.1B may affect cell proliferation by regulating EGFR. The finding that EGFR inhibitor erlotinib inhibited the proliferation of 4.1B KO MEFs (Fig. 5C) supports this hypothesis. To investigate the mechanisms for the increased EGFR protein level, we examined the mRNA levels of EGFR in 4.1B-sufficient and 4.1B-deficient cells by real time PCR. Figure 5D shows negative correlation between 4.1B expression and EGFR mRNA levels in various cell types, demonstrating that deficiency of 4.1B leads to increased EGFR transcription.

To further define mechanisms for the increased mRNA level of EGFR, we examined the expression of Sp1, the transcription factor for EGFR. The data in Fig. 5E shown that 4.1B deficiency led to increased expression of Sp1. Interestingly, knockdown of Sp1 in 4.1B-deficient cells led to decreased EGFR expression at mRNA and protein level (Fig. 5F).

Given the consistency in the data between MEFs and GC cell lines, we used MEFs as the experimental model in subsequent assays.

### Association of 4.1B with EGFR in MEF cells

**Fig. 6:**
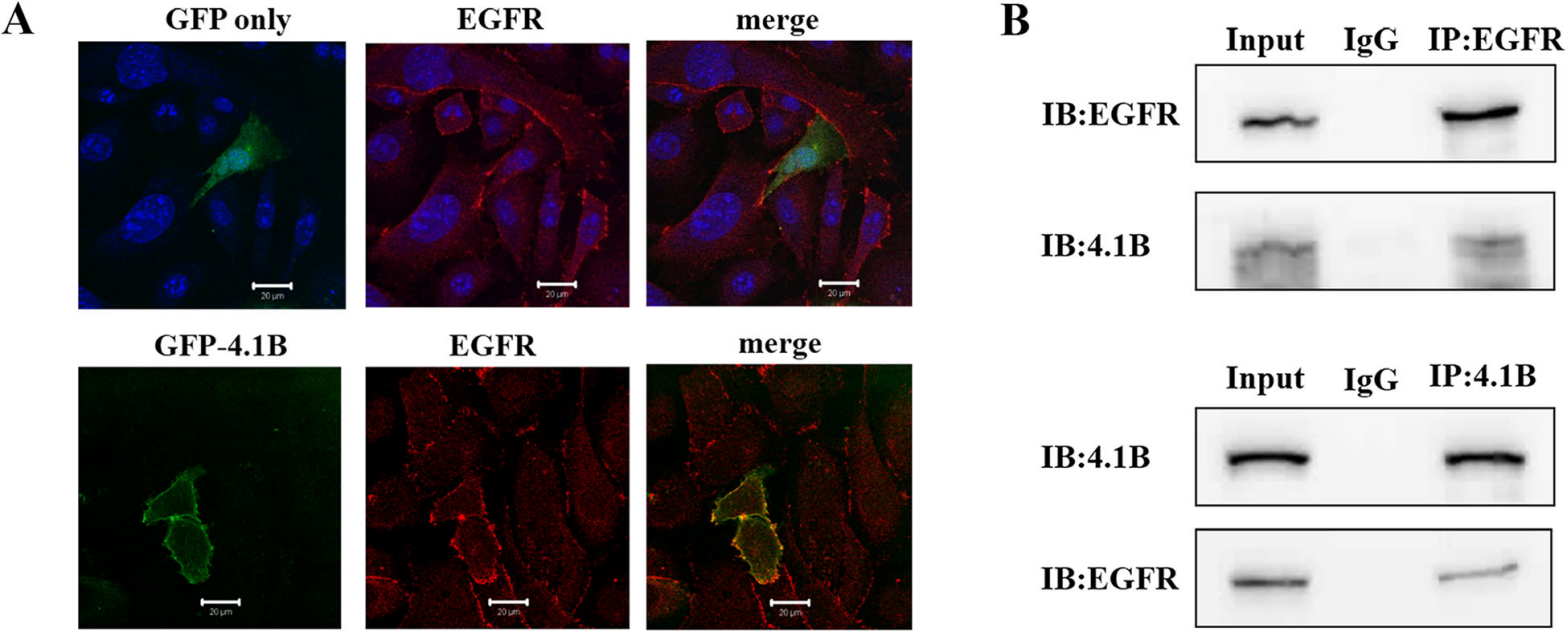
Interaction of 4.1B with EGFR in immortalized MEF cells. **a** 4.1B co-localization with EGFR in immortalized WT MEF by IF. Scale bars, 20 μm. **b** 4.1B binds to EGFR by Co-IP

We next examined the association of 4.1B with EGFR in MEF cells. Figure 6a shows that while GFP was diffusely located in the cytosol, GFP-4.1B co-localized with EGFR at the plasma membrane. Moreover, Fig. 6b shows that endogenous EGFR was pulled down by an anti-4.1B HP antibody and that endogenous 4.1B was pulled down by an anti-EGFR antibody. These findings suggest that 4.1B binds to EGFR.

### 4.1B FERM domain binds to EGFR JM segments

**Fig. 7:**
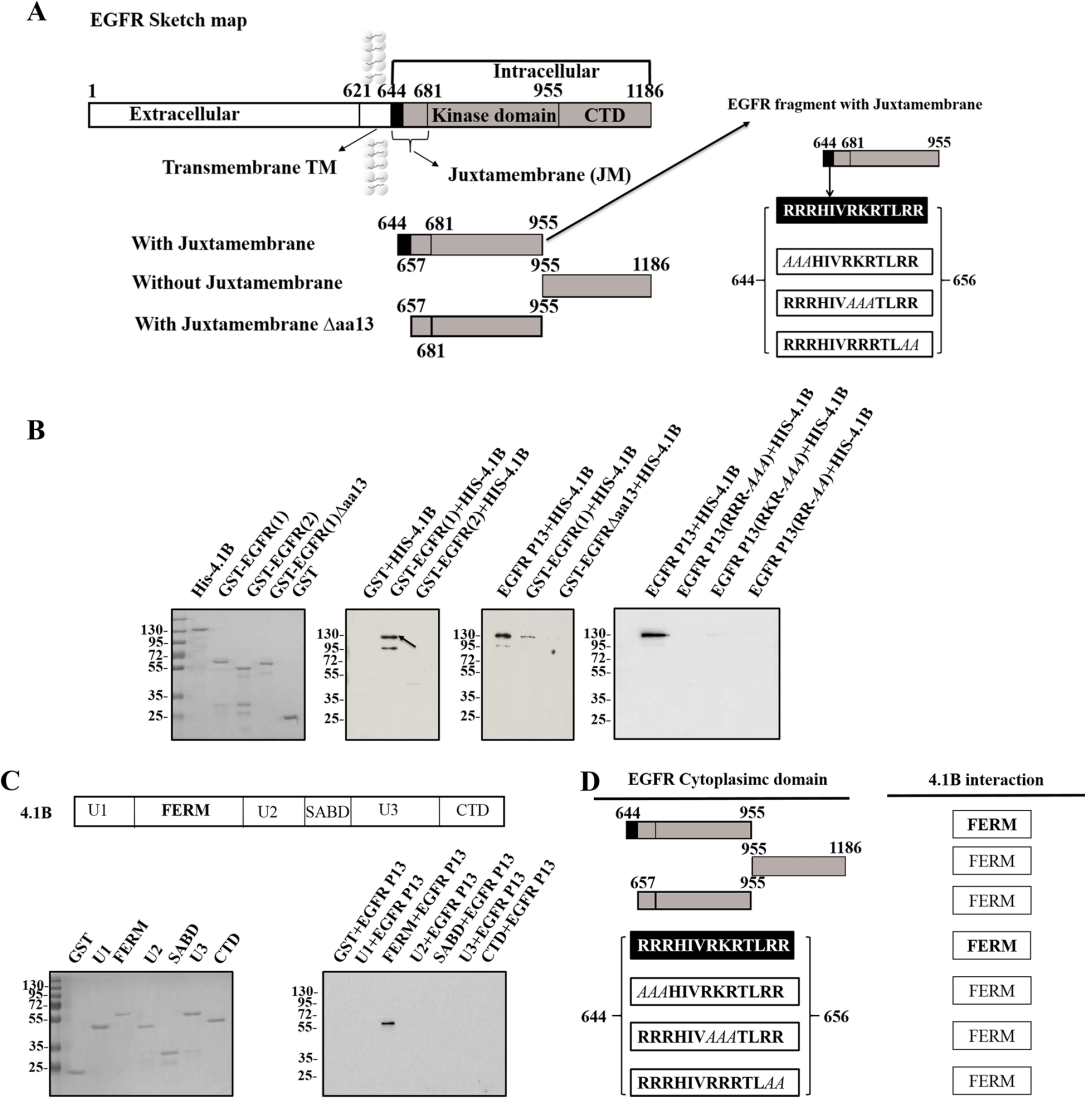
4.1B FERM domain binds to EGFR JM segments. **a** Sketch map of intracellular EGFR design information. GST-tagged three fragments of intracellular EGFR: EGFR fragment with (amino acids fragment 644–955 (EGFR1)) and without (amino acids fragment 956–1186 (EGFR2)) JM fragments and JM fragment deletion the first thirteen amino acids (amino acids fragment 657–955 (EGFR1△aa13)). The black arrow shows EGFR fragment (amino acids fragment 644–955 (EGFR1)) with two or three amino acids mutated randomly: aa13-RRRHIVRKRTLRR, EGFR P13(RRR-*AAA*)-*AAA*HIVRKRTLRR, EGFR P13(RKR-*AAA*)- RRRHIV*AAA*TLRR, EGFR P13(RR-*AA*)-RRRHIVRRRTL*AA*. *AAA* means mutated region. **b** 4.1B binds to EGFR fragments with the first thirteen amino acids of JM fragment by pull-down assay. **c** Sketch map of domains of 4.1B and the FERM domain of 4.1B binds to EGFR by pull-down assay. **d** Conclusions of 4.1B FERM domain interacts with EGFR fragment with the first thirteen key amino acids of JM fragment

To further characterize the direct binding between 4.1B and EGFR, we performed a GST pull-down assay using 3 different GST-tagged EGFR intracellular fragments: fragment with JM region (amino acids fragment 644–955 (EGFR1)), fragment without (amino acids fragment 956–1186 (EGFR2)) JM region, and fragment with JM fragment with the first 13 amino acids deleted (EGFR1△aa13) (amino acids fragment 644–955) (Fig. 7a).

We found that 4.1B interacted with EGFR JM fragment with the first 13 key amino acids (Fig. 7b). The P13 sequence of EGFR JM segment is “**RRRHIVRKRTLRR”** (aa13). We designed three biotin-tagged P13 mutated fragments (*AAA***HIVRKRTLRR** (EGFR P13(RRR-*AAA*)), **RRRHIV***AAA***TLRR** (EGFR P13(RKR-*AAA*), and **RRRHIVRRRTL***AA* (EGFR P13(RR-*AA*)) (Fig. 7a). Binding was eliminated when two or three amino acids were randomly mutated (Fig. 7b). We also performed a GST pull-down assay using GST-tagged 4.1B domains. The FERM domain of 4.1B was found to bind with EGFR intact JM fragment (Fig. 7c). In summary, we have defined the binding sites involved in 4.1B-EGFR interaction. This interaction is schematically represented in Fig. 7d.

## Discussion

The results of our study have highlighted a novel function for 4.1B as a tumor suppressor, which inhibits proliferation of gastric cancer cells. Additionally, we demonstrate the details of the sites involved in interaction between 4.1B and EGFR. This study is the first to document the mechanism by which 4.1B inhibits tumor cell proliferation by binding to the JM segment of EGFR.

Previous studies showed that different cancers express little to no 4.1B [14–17] due to promoter hypermethylation [14, 19, 34–36]. Only a few studies have assessed 4.1B in GC [18, 19]. Our study examined the correlation of 4.1B with GC clinical parameters. The IHC results showed that 4.1B was mainly expressed in the cell plasma membrane and was downregulated in GC adenocarcinoma. Our clinical data also demonstrated that 4.1B can affect tumor size, pathologic differentiation, vascular invasion, and TNM stage. The expression of 4.1B is also associated with patient overall survival (OS). These results suggest that 4.1B may serve as a tumor suppressor in gastric cancer.

Several studies have alluded to the molecular mechanisms underlying the oncogenic effect of 4.1B deficiency. Sakurai-Yageta et al. reported that 4.1B regulated CADM1 to affect epithelial cell adhesion. CADM1 associates with 4.1B/DAL-1 through the 4.1B binding motif, while loss of the CADM1–4.1B/DAL-1 complex may affect cancer cell adhesion and enhance cancer cell invasion and/or metastasis [30]. Other studies have shown that 4.1B/DAL-1 can anchor F-actin to the cell membrane, thereby inhibiting cell motility by supporting orderly arrangement of actin stress fibers. Loss of the 4.1B/DAL-1 complex may promote F-actin reorganization and enhance cell motility, which in turn, may increase tumor migration [15]. Other studies have also reported 4.1B regulation of cell mobility through the activity of adhesion molecules [15, 30, 37, 38].

In this study, we focused on the functional role of 4.1B in gastric cancer cell proliferation. Although studies have examined the relationship of 4.1B and GC [18, 19], there have been no reports of the functional effect of 4.1B on GC cell proliferation. We investigated the relationship between protein 4.1B and proliferation and tumorigenicity using pre-clinical in vitro and in vivo models of GC. Our results showed that 4.1B can inhibit tumor cell proliferation. Robb et al., Gerber et al., and Kuns et al. have reported that the U2 domain of 4.1B can affect cell proliferation by activating Src, Rac1, MLK3, and JNK, resulting in reduced expression cyclin A and decreased retinoblastoma (Rb) protein hyperphosphorylation [33, 39, 40].

We found that 4.1B blocked GC cell line proliferation through the proto-oncogenic EGFR/MAPK/ERK1/2 and PI3K/AKT signaling pathways. Of note, we observed that perturbing 4.1B expression in GC cell lines resulted in a change in the levels of total EGFR. Similar effects on proliferation and EGFR levels were observed in immortalized 4.1B deficiency MEFs.

We used an EGFR inhibitor to address our hypothesis that 4.1B can inhibit EGFR-mediated cell proliferation in immortalized 4.1B deficiency MEFs. Our results also showed that 4.1B can suppress EGFR mRNA levels.

Activation of EGFR requires more than just ligand engagement to the extracellular EGFR segment. EGFR dimerization also requires coupling of the transmembrane domain and JM segment [41]. The initial 13 amino acids (P13) within the intracellular JM region (R645–R657) are required for proper dimerization and autophosphorylation of EGFR [42]. 4.1B is localized to the plasma membrane and is distributed along cell–cell junctions [26], as well as cell-basement membrane contacts [33]. Therefore, we hypothesized that 4.1B could bind to the P13 region of the EGFR JM segment to block EGFR activation. Our IF staining and co-IP assay demonstrated that 4.1B can indeed, directly bind to EGFR. Our pull-down assay clearly showed 4.1B interaction with the P13 region of the EGFR JM fragment. We further demonstrated that the FERM domain of 4.1B can bind to the P13 region of the EGFR JM segment.

**Fig. 8:**
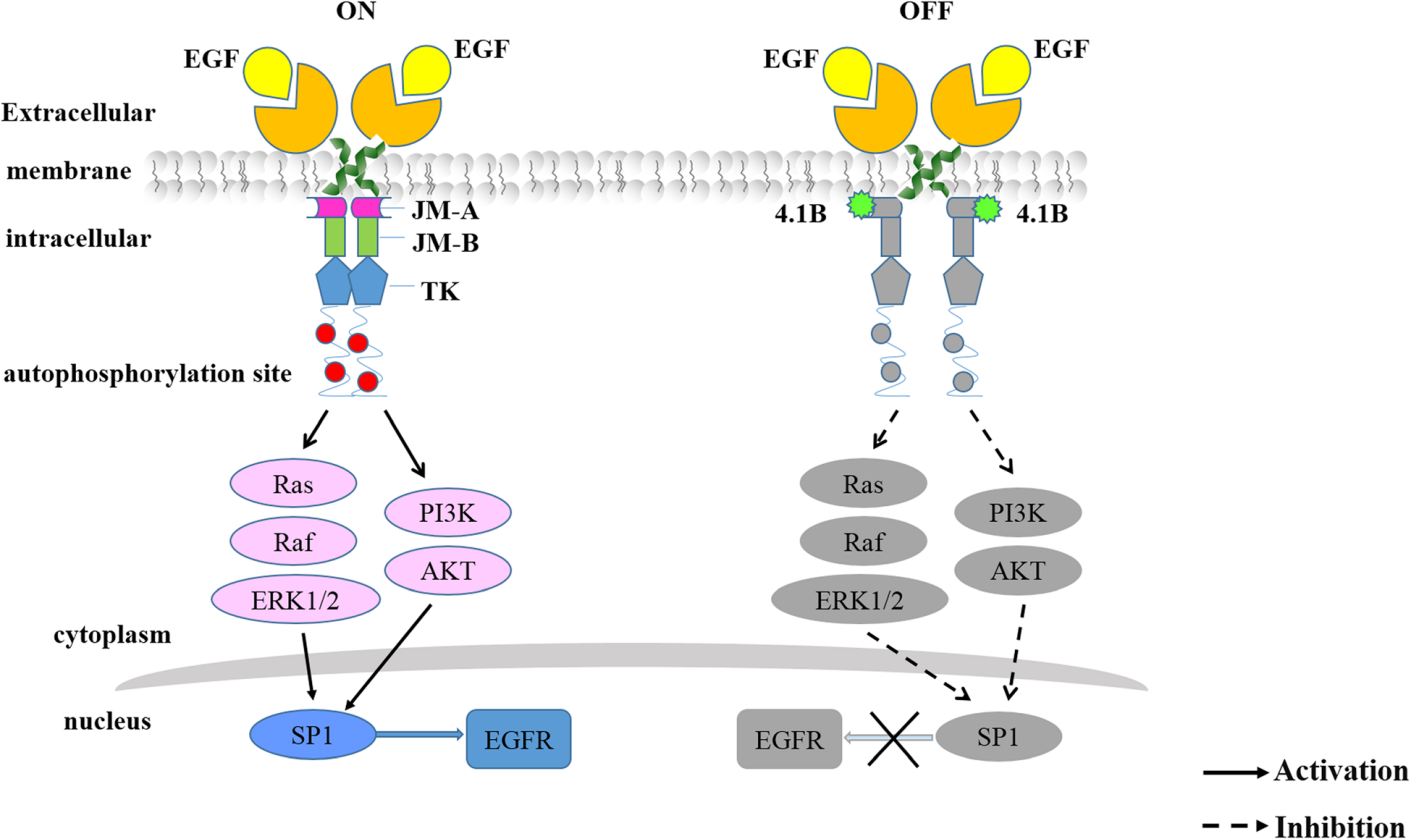
Schematic presentation of the mechanisms by which 4.1B inhibits the activation of EGFR

Our schematic diagram (Fig. 8) explains clearly the mechanism of 4.1B blocking cells proliferation by inhibiting the EGFR synthesis. The combination of protein 4.1B with P13 of EGFR JM segments blocks the binding of EGFR monomers intracellular JM segments. This blocking hinders EGFR monomers dimerization and autophosphorylation, and the conformational coupling is stopped. Thus, EGFR activation and autophosphorylation is suppressed in 4.1B-sufficient cancer cells. This can explain why the phosphorylated EGFR was down-regulated in our transfected GC cells and MEF cells with protein 4.1B existence. The data showed that phosphorylated ERK1/2 was down-regulated by inhibition of EGFR/Ras/Raf/ERK1/2 pathway.

## Conclusion

Our findings are significant and novel. First, our results clearly showed that 4.1B functions as a tumor suppressor in GC. Our study is also the first report of 4.1B inhibition of GC cell proliferation by binding to EGFR monomers. This is also the first demonstration of the precise domains of 4.1B and EGFR that facilitate their interaction. Given the importance of EGFR signaling in cancer cell growth and tumor progression, our study highlights a potential new therapeutic target for gastric cancer. Our data suggest that 4.1B-targeting may be a viable strategy to combat tumor progression in gastric cancer patients.

## Data Availability

The datasets used and/or analysed during the current study are available from the corresponding author upon request. All data generated or analysed during this study are included in this published article.

## Abbreviations

AJCC: American Joint Committee on Cancer
BKO: 4.1B knock-out
Co-IP: Co-immunoprecipitation
DFS: Disease-free survival
DMEM: Dulbecco Modified Eagle Medium
EGFR: Epidermal growth factor receptor
FBS: Fetal bovine serum
GC: Gastric cancer
HE: Hematoxylin and eosin
IF: Immunofluorescence
JM: Juxtamembrane
MEF: Mouse embryonic fibroblasts
OS: Overall survival
P13: Initial 13 amino acids
Sp1: Specificity protein 1
SPF: Specific pathogen-free
WT: Wild type
